# Development of a Sterne-Based Complement Fixation Test to Monitor the Humoral Response Induced by Anthrax Vaccines

**DOI:** 10.3389/fmicb.2016.00019

**Published:** 2016-01-28

**Authors:** Rosanna Adone, Michela Sali, Massimiliano Francia, Michela Iatarola, Adelia Donatiello, Antonio Fasanella

**Affiliations:** ^1^Istituto Superiore di SanitàRome, Italy; ^2^Università Cattolica del Sacro CuoreRome, Italy; ^3^Istituto Zooprofilattico Sperimentale della Puglia e Basilicata, National Reference Centre for AnthraxFoggia, Italy

**Keywords:** anthrax, serology, complement fixation test, antibody response, vaccination

## Abstract

Anthrax is a zoonotic disease caused by *Bacillus anthracis* spore-forming bacterium. Since it is primarily a disease of animals, the control in animals, and humans depend on the prevention in livestock, principally cattle, sheep, and goats. Most veterinary vaccines utilize the toxigenic, uncapsulated (pXO1+/pXO2–) *B. anthracis* strain 34F_2_ which affords protection through the production of neutralizing antibodies directed to the toxin components Protective Antigen (PA), Lethal Factor (LF), and Edema Factor (EF). The titration of specific antibodies in sera of vaccinated animals is crucial to evaluate the efficacy of the vaccination and to obtain epidemiological information for an effective anthrax surveillance. In this study, we developed a Sterne-based Complement Fixation Test (CFT) to detect specific antibodies induced in animals vaccinated with Sterne 34F_2_. We assessed its efficacy in laboratory animals and under field conditions by monitoring the humoral response induced by vaccination in cattle. The results indicated that the Sterne-based CFT is able to correctly identify vaccinated animals. It proved to be a very sensitive and specific test. Moreover, the Sterne-based CFT offers many benefits with regard to costs, standardization and reproducibility of the assay procedure.

## Introduction

Anthrax is a zoonotic disease with an almost worldwide distribution, with prevalence in tropical and sub-tropical countries (Biswas et al., 2011). The causative agent is *Bacillus anthracis*, a Gram-positive, aerobic, spore-forming bacterium. It is primarily a disease of herbivores; ruminants such as cattle, goats, and sheep are the most susceptible animals (World Health Organization [WHO], 2008). Animals become infected thorough contact with soil contaminated with persistent bacterial spores. Humans are susceptible to infection through contact with infected animals or animal products contaminated with anthrax spores. Depending of the route of the exposure, human anthrax infection can take three forms: cutaneous, gastro-intestinal, and inhalational anthrax. The majority of reported cases are cutaneous (95%), especially in developing countries whose main source of income is farming (Ghosh and Goel, 2012).

*Bacillus anthracis* has two principal virulence factors, the toxin complex, and the poly-γ-D-glutamic acid capsule, coded by plasmids pXO1 and pXO2, respectively. The capsule protects the bacterium from phagocytosis while the toxin complex consists of three synergistically acting proteins, Protective Antigen (PA), Lethal Factor (LF), and Edema Factor (EF), produced during the log phase of growth. The interaction of these proteins forms the lethal and edema toxins, responsible for cytotoxic effects (Collier and Young, 2003; World Health Organization [WHO], 2008). The protection against anthrax mainly depends on the host’s humoral response to the toxin components (Turnbull et al., 1988; Pitt et al., 2001; Friedlander et al., 2002). The PA is a key element: the anti-PA antibodies are not, in themselves, a guarantee of protected status, but they must be in the blood of animal or human for the subject to be protected (Ivins and Welkos, 1988; Ivins et al., 1990; Little et al., 1997).

Since anthrax is primarily a disease of animals, its control in animals and humans depends on the prevention in livestock, principally cattle, sheep, and goats. Most anthrax vaccines for animals utilize the *B. anthracis* strain 34F_2_, developed by Max Sterne in 1937 (Sterne, 1939) which lacks genes for capsule formation but still produces the toxin (pXO1+/pXO2–), responsible for the induction of protective antibodies. The protective effect of a single dose of this vaccine is said to last for 9 to 12 months so annual boosters are recommended in endemic areas (Sterne, 1939). Also, a single dose of Sterne vaccine may not be sufficient to ensure protective immunity in the animal to last for a year, and more than one initial dose of the Sterne vaccine may be necessary (Turnbull et al., 2004; Mongoh et al., 2008; World Health Organization [WHO], 2008). However, very few studies focusing on immunological characterization have been conducted with cattle and goats (Dipti et al., 2013; Roy et al., 2013).

Serological methods, which have not a prominent role in diagnosis, are very useful epidemiological and research tools (Turnbull et al., 1992; Quinn et al., 2004) since they enable to monitor the humoral response following vaccination or naturally acquired infection. The titration of specific antibodies in sera of vaccinated animals is crucial to evaluate the efficacy of the vaccination and to obtain epidemiological information in areas where the disease is endemic.

Currently, the ELISA is considered the most effective serological method; however, it utilizes purified toxin antigens PA and LF whose preparation is expensive and lacks of a good standardization in regard to purity, composition of antigens and preparation procedure. Moreover, reference standard serum is not available.

The Complement Fixation Test (CFT) is a serological method prescribed as individual confirmatory test in many infectious diseases. It is able to detect specific antibodies in animal and human serum samples and may detect incomplete antibodies. The antigen preparation, mainly consisting in an inactivated bacterial suspension, is not expensive and can be easily standardized.

The purpose of this study was to develop a Sterne-based CFT able to detect anti-anthrax antibodies induced in animals vaccinated with the Sterne 34F_2_ vaccine. We assessed the efficacy of our method in laboratory animals and under field conditions by monitoring the humoral response induced in cattle by vaccination with Sterne 34F_2_.

## Materials and Methods

### Bacterial Strains and Growth Conditions

The *B. anthracis* strain 34F_2_, used for the preparation of the veterinary vaccine, and the virulent strain *B. anthracis* A0843 (Fasanella et al., 2001) were supplied by the National Reference Centre for Anthrax (Ce.R.N.A.) of the Istituto Zooprofilattico Sperimentale of Puglia and Basilicata (Foggia, Italy). This Institute is charged with the production of the Sterne 34F_2_ vaccine, routinely used for immunization of susceptible animal species in Italy (Italian DM 7-7-1992). Both strains were cultured at 37°C on agar tryptose soy agar supplemented with 5% of equine serum (TSA/S).

### Animals

In this study the ability of the Sterne-based CFT to detect the antibody response induced by vaccination with Sterne 34F_2_ was evaluated in rabbits and cattle.

New Zealand White (NZW) rabbits, weighting 1.2–1.5 kg, provided by Harlan Laboratories srl (Udine, Italy) were individually housed in barrier housing with filtered inflow air in a restricted-access room and under pathogen-limited conditions. Experiments were conducted in accordance with the current European Legislation (Directive 86/609/EEC) relating the welfare of animals involved in experiments.

Breed Limousine female cattle, 10–12 months of age, belonging to a farm in Basilicata, a southern region of Italy where anthrax is enzootic, were used.

### Experimental Design

Thirty-eight NZW rabbits were vaccinated subcutaneously (s.c.) two times, at 15 days interval, with 1 ml/each containing 1.2 × 10^7^ live spores of Sterne 34F_2_ vaccine. Blood samples were collected from all rabbits prior to vaccination (day 0), 15 days after the first vaccination (15 days post-vaccination, dpv) and 15 days after the second vaccination (30 dpv). Nine unvaccinated rabbits were kept as controls.

On the same day of the last sampling, all rabbits, vaccinated and controls were challenged with 200LD_50_ of *B. anthracis* virulent strain A0843 to evaluate the protective activity of the vaccine.

Forty-six female cattle were vaccinated subcutaneously with 1 ml/each containing 1.2 × 10^7^ live spores of Sterne 34F_2_ vaccine, as prescribed by the Italian DM 7-7-1992. Ten, unvaccinated female cattle belonging to the same farm were kept as controls.

Blood samples were collected prior vaccination (day 0) and then at 12, 30, and 150 dpv.

After incubation at 37°C for 30 min to clot, serum samples were collected by centrifugation and stored at –20°C until use. All sera were tested by the Sterne-based CFT.

### Complement Fixation Test: Principle of the Method

The CFT consists of two stages: in the first stage, specific antigen and test serum are mixed with guinea-pig serum which contains the complement. If specific antibodies are present in the test serum, the complement combines with the antigen–antibody complex and cannot react in the second stage of the reaction. In the second stage, sheep erythrocytes previously sensitized with rabbit serum anti-sheep erythrocytes (hemolytic system), are added. If the complement has been fixed in the first stage, no hemolysis will occur (positive reaction). On the contrary, if the serum test doesn’t contain specific antibodies, the complement, not consumed, can combine with the hemolytic system causing the lysis of sheep erythrocytes (negative reaction).

The CFT required the following reagents: (i) lyophilized guinea-pig complement (serum of healthy guinea pigs); (ii) hemolysin stock solution (monoclonal antibodies against sheep erythrocytes), (iii) sheep erythrocytes (54 × 10^7^ cells/ml); (iv) bovine, hyperimmune serum containing anti-anthrax antibodies (positive control); (v) pool of sera from healthy, unvaccinated cattle (negative control).

For this study, sheep erythrocytes, hemolysin, and complement were supplied by Emozoo Snc of Ripabelli G. & C. (Siena, Italy). Positive and negative controls, previously evaluated by ELISA (Fasanella et al., 2007), were provided by the Ce.R.N.A.

The Veronal calcium–magnesium buffer, pH 7.2 ± 0.1 (Lonza, Walkersville, Inc., USA), added with 0.1% of bovine serum albumin (VBA), was used as diluent.

### Sterne Antigen Preparation and Titration for Use in CFT

The Sterne antigen for CFT was prepared as follows: single colonies of *B. anthracis* strain 34F_2_ were cultured for 24 h at 37°C on TSA/S plates and then on Roux flasks for propagation. After incubation, bacteria were harvested with physiological saline (pH 7.2), inspected for purity by Gram staining and washed twice by centrifugation at 1.430 g for 15 min. For the inactivation, the bacterial suspension was added with 3% of formalin saline and incubated overnight at 37°C. After inactivation checking, the suspension was centrifuged as above and the pellet was suspended in 0.5% of formalin saline for storage until use.

For use in the CFT, the Sterne antigen was titrated as prescribed by the OIE Manual of Diagnostic Tests for diagnosis of brucellosis (Office International des Epizooties [OIE], 2008). Briefly, doubling dilutions of the antigen, each supplemented with 5% of Fetal Calf Serum (FCS, OXOID), were tested in CFT to establish the optimal concentration which was able to: (i) react positively with the positive control; (ii) do not react with the negative control; (iii) do not show anticomplementary activity. The anticomplementary activity occurs when the antigen fixes the complement in absence of the antigen-antibody complex, thus giving false positive reactions.

The optical density of the optimal antigen concentration, measured at 490 nm, gave a value of 0.2.

### Sterne-Based CFT on Rabbit and Bovine Serum Samples

Before the CFT, all sera were diluted 1:2 in VBA and incubated for 30 min at the appropriate temperature to inactivate the native complement (58°C for cattle, 62°C for rabbits). The Sterne-based CFT was performed as previously described (Adone and Ciuchini, 1999; Adone et al., 2008). Briefly, in 96-well round-bottom plates 25 μl of each serum was serially diluted in VBA from 1:2 to 1:128. Then, 25 μl of antigen and 25 μl of complement, at working dilution, were added and plates were incubated at 37°C for 30 min. After incubation, 25 μl of sensitized erythrocytes were added to each well. After incubation as above, plates were centrifuged at 200 g for 5 min to allow any unlysated cells to deposit and the reaction was read over a diffused white light. The results were evaluated as follows: 100% hemolysis was considered as negative reaction while all reactions showing complete absence of hemolysis (0%) or partial hemolysis 75, 50, or 25% were considered as positive. The titer of each serum was the highest dilution showing a positive reaction. Based on our previous experience (unpublished data), the serum dilution 1:2 showing 50% of hemolysis was taken as the reactivity threshold of the reaction.

### Sensitivity (SN) and Specificity (SP) of the Sterne-Based CFT

The SN and SP of the Sterne-based CFT were determined, at different times, by categorizing the results into reactive and non-reactive by application of the reactivity threshold (titer 1:2). The SP of the assay was calculated as TN/(TN+FP), where TN = true negatives and FP = false positives. The SN of the assay was calculated as TP/(TP+FN), where TP = true positives and FN = false negatives.

Sterne-vaccinated animals were considered as true positives, while unvaccinated controls and animals bled prior vaccination were considered as true negatives.

### Statistical Analysis

Different percentage of serology performed at different time points was calculated by using Contingency tables for non-parametric data (Fisher’s exact test). Results were considered significant at *p* ≤ 0.05.

## Results

### Rabbit Sera CFT Titers

**FIGURE 1:**
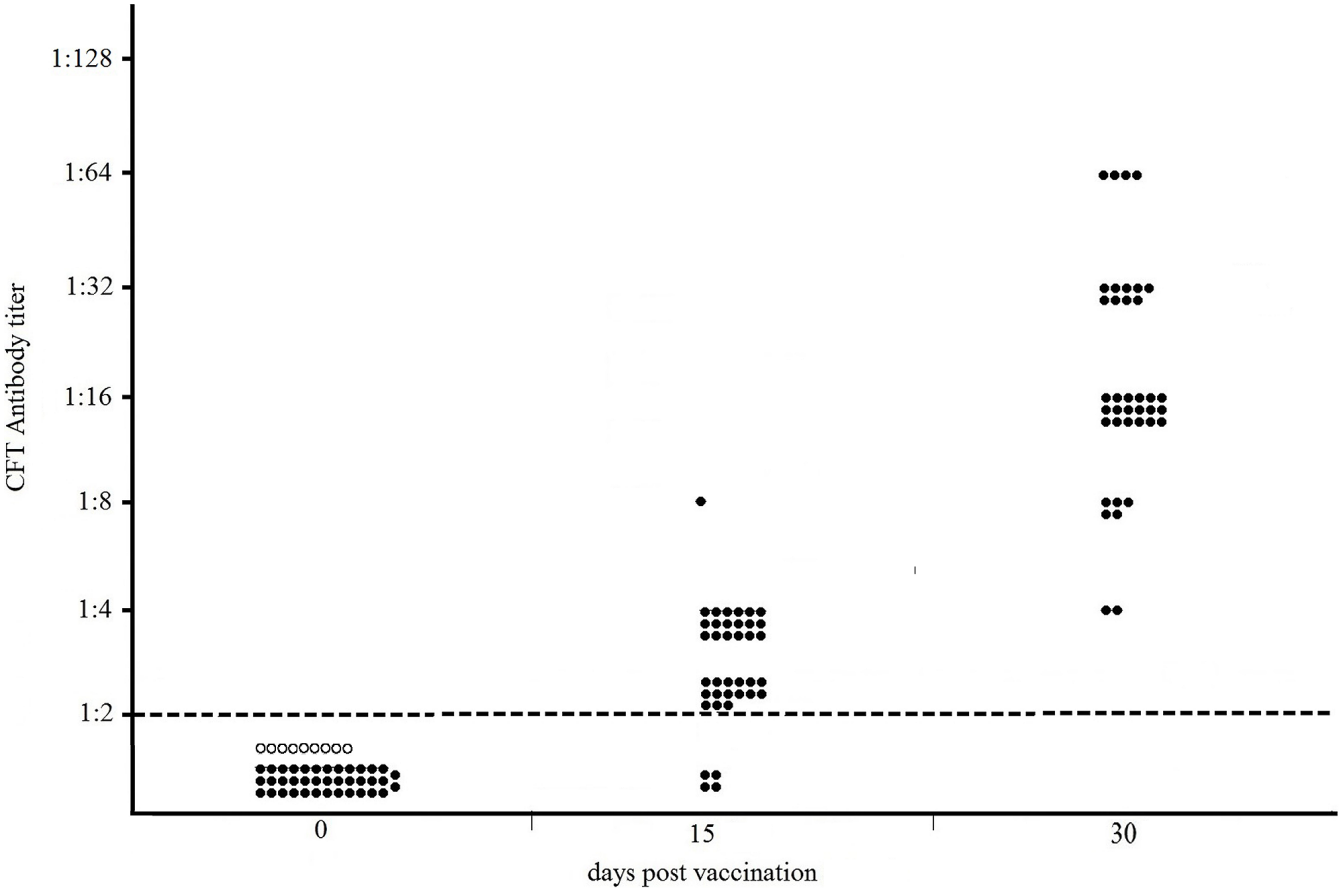
**Distribution of CFT titers of thirty-eight NZW rabbits vaccinated with 1.2 × 10^7^ live spores of Sterne 34F_2_/each, measured by Sterne-based CFT prior to vaccination (dpv 0), at 15 and 30 days post vaccination.** At 15 dpv, rabbits received the second dose of vaccine. Black and white circles represent the vaccinated and unvaccinated (controls) rabbits, respectively. The broken line indicates the reactivity threshold of the reaction (titer 1:2).

The results of the Sterne-based CFT performed on sera collected from 38 NZW rabbits vaccinated with Sterne 34F_2_ (1.2 × 10^7^ live spores /each), are showed in **Figure 1**. As shown, none of serum samples from rabbits bled prior vaccination (dpv 0) and from nine unvaccinated controls gave positive results. At 15 days after the first vaccination (15 dpv), 89% (34/38) of rabbits weakly reacted in CFT, titers ranging from 1:2 (15/38), considered as the reactivity threshold of the reaction, to 1:8 (1/38). The majority of them, (18/38), gave a titer of 1:4. In contrast, at 15 days after the second vaccination (30 dpv), all rabbits (38/38) were seropositive with titers ranging from 1:4 (2/38) to 1:64 (4/38), with a prevalence of titer 1:16 (18/38). Five rabbits (5/38) titrated 1:8, and nine rabbits (9/38) gave a titer 1:32. All CFT titers were significantly over the reactivity threshold (Fisher’s exact test *p* < 0.01).

All seropositive rabbits survived to the challenge with the virulent strain while all controls died (data not shown).

### Bovine Sera CFT Titers

**FIGURE 2:**
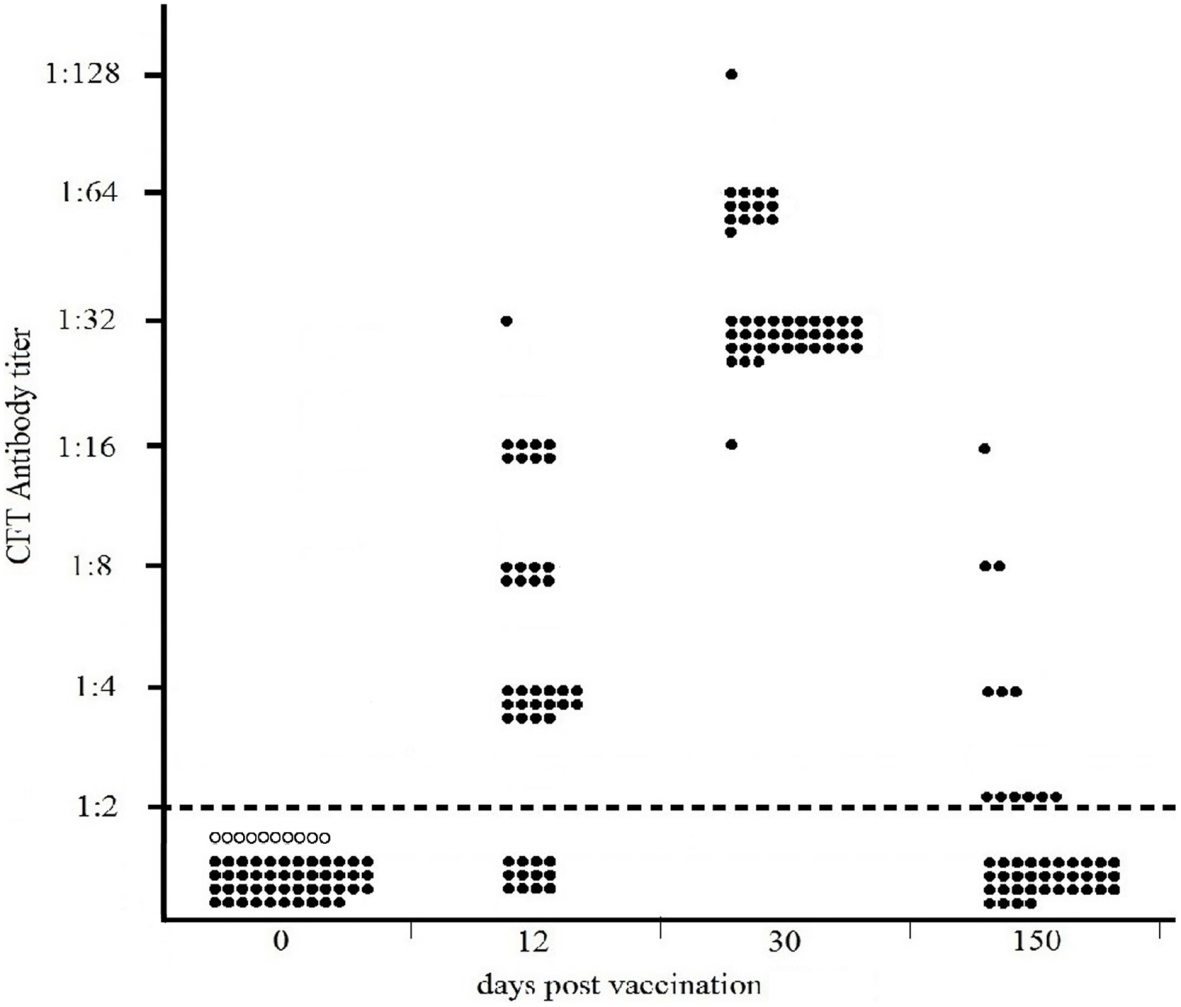
**Distribution of CFT titers of forty-six cattle vaccinated with 1.2 × 10^7^ live spores of Sterne 34F_2_/each, measured by Sterne-based CFT prior to vaccination (dpv 0) and at 12, 30, and 150 dpv.** Black and white circles represent the vaccinated and unvaccinated (controls) cattle, respectively. The broken line indicates the reactivity threshold of the reaction (titer 1:2).

The results of the Sterne-based CFT performed on sera collected from 46 cattle vaccinated with Sterne 34F_2_ (1.2 × 10^7^ live spores /each), are presented in **Figure 2**. As shown, none of cattle bled prior to vaccination (dpv 0) or unvaccinated controls were seropositive, thus indicating 100% of specificity. At 12 days post vaccination, complement fixating antibodies to Sterne were detected in 72% of calves (33/46), titers ranging from 1:4 (16/46) to 1:32 (1/46). Eight cattle (8/46) gave a titer 1:8 and the remaining eight cattle (8/46) gave a titer 1:16. At 30 dpv, all cattle (46/46) were seropositive, titers significantly increasing from 1:16 (1/46) to 1:128 (1/46). Most of them (31/46) gave a titer 1:32, while 13/46 titrated 1:64. All CFT titers were significantly over the reactivity threshold. At 150 dpv, only 13% (6/46) of calves were still seropositive (Fisher’s exact test *p* = 0.02) with decreased titers from 1:4 (3/46) to 1:16 (1/46). Two cattle (2/46) titrated 1:8. Six cattle (6/46) gave a titer 1:2 corresponding to the reactivity threshold of the reaction.

### Kinetic of the Antibody Response Induced by Vaccination in Cattle

As shown in **Figure 2**, at 12 days post vaccination, detectable antibodies to Sterne were present in 72% of cattle (33/46). At 30 dpv, 100% of cattle were seropositive, showing high and similar antibody titers to Sterne antigen. At 150 dpv, specific antibodies to Sterne were detected only in 13% of cattle.

## Discussion

The protective activity of anthrax vaccines mainly depends on their ability to elicit antibodies directed to toxin components (Little et al., 1997; Beedham et al., 2001; Reuveny et al., 2001; Kobiler et al., 2002); however, little is known on the effective duration of immunity, many studies demonstrating the decline of the specific antibody response and the need of different vaccination schedules to ensure protection. New, improved livestock vaccines should be developed able to induce high level of protective antibodies in a very short time and that could be administered with long-acting antibiotics during the anthrax outbreaks or in emergency (Welkos et al., 2001; Fasanella et al., 2008; World Health Organization [WHO], 2008).

For humans, licensed vaccines are composed by killed, cell-free suspensions, to avoid the risks due to the residual virulence of live strains. They are targeted at persons occupationally exposed to anthrax spores or involved in occupations related to defense.

In contrast, most veterinary vaccines are composed by live spores of attenuated (non-capsulated) *B. anthracis* strain 34F_2_ (Sterne, 1939) suspended in saponin for enhancing immune response. The non-capsulated variants of *B. anthracis* may lose their immunogenicity when cultured, so their efficiency must be monitored (Sterne, 1939, 1959).

Serological tests, which have not a great diagnostic value, proved to be a very useful epidemiological and research tool to evaluate the seroconversion following vaccination or naturally acquired infection (Turnbull et al., 1992; Quinn et al., 2004). Demonstration of specific antibodies in sera of vaccinated animals is needed to obtain epidemiological information in areas where the disease is endemic and to evaluate the efficacy of the vaccination. In effect, there is still a general paucity of information related to the onset, kinetic, and magnitude of antibody response induced by vaccination in humans and animals.

Effective serological tests should be satisfactory in terms of sensitivity and specificity, not expensive, to avoid that their use is confined to a few specialist laboratories, and easy to standardize.

In this study, we assessed the suitability of a Sterne-based CFT as method for the detection of specific antibodies in laboratory and target animals vaccinated with Sterne 34F_2_. We evaluated its specificity and sensitivity by testing unvaccinated and vaccinated animals, respectively, at different sampling times.

We prepared and titrated an inactivated Sterne suspension for use as antigen in CFT according to OIE indications and our CFT experience (Adone and Ciuchini, 1999; Adone et al., 2008). The addition of FCS increases the sensitivity of the reaction, reducing the number of false negative results as previously suggested (Cho, 1971).

The humoral response in NZW rabbits vaccinated with Sterne 34F_2_ was evaluated according to the procedure used by Ce.R.N.A. to measure the potency of anthrax vaccines. For this purpose, rabbits were vaccinated twice, 15 days apart, with 1.2 × 10^7^ live spores of Sterne 34F_2_ and 15 days after the second vaccination all rabbits, vaccinated and controls, were challenged with 200LD_50_ of the virulent strain *B. anthracis* A0843. The antibody response was monitored prior to vaccination, 15 days after the first vaccination, and 15 days after the second vaccination, on the same day of the challenge. As shown in **Figure 1**, the Sterne-based CFT was able to detect all vaccinated rabbits at 30 dpv, 15 days after the second vaccination. Most of seropositive rabbits gave complement fixating titers ranging from 1:16 to 1:32 thus significantly over the reactivity threshold. All seropositive rabbits survived to the challenge with the virulent strain while all controls died (data not shown) indicating that the humoral response measured by the Sterne-based CFT was protective. None of serum samples collected from rabbits prior vaccination or from unvaccinated controls gave positive results, thus indicating 100% of specificity (**Figure 1**).

Data obtained in rabbits indicated that a correlation between the complement fixating titers to Sterne and the protective activity afforded by the vaccine may be experimentally established. Based on our results, rabbits showing detectable complement fixating antibodies with titers significantly over the threshold of 1:2 were protected against anthrax infection.

Additional tests are in progress in our laboratory to confirm the suitability of the Sterne-based CFT as tool to measure the potency of anthrax vaccines in laboratory animals by evaluating antibody response in replacement of the challenge with virulent strains. The availability of methods alternative to experimental infection could avoid the need of containment equipment and facilities required to reduce the risks for personnel exposed to biosafety level 3 agents (Little et al., 2004). Moreover, it could minimize animal suffering, in agreement with principles for more ethical use of animals.

In cattle, the Sterne-based CFT was able to detect vaccinated animals with a sensitivity ranging from 72% at 12 dpv to 100% one month after the vaccination (**Figure 2**). At this time, complement fixating titers ranged from 1:16 to 1:128 with a prevalence of titer 1:32 thus significantly over the reactivity threshold. At 150 dpv, only 13% of cattle were still seropositive, showing weak CFT titers. None of cattle bled prior vaccination or unvaccinated cattle gave positive results, thus confirming the high specificity of this test (**Figure 2**).

To evaluate the ability of the Sterne-based CFT to detect antibodies in other species, we tested serum samples from Sterne-vaccinated sheep, goats and horses: all vaccinated animals were identified, while proven uninfected or unvaccinated animals did not react (data not shown). Continuing validation is ongoing as samples with proven status and provenance become available.

As a serological method, the Sterne-based CFT could offer many benefits: (i) the reagents are not expensive and may be easily standardized: the optimal concentration of the antigen may be accurately determined and standardized. The other reagents are titrated according to classical procedures described by the OIE; (ii) the preparation of the antigen does not require particular growth conditions or safety measures since risks to personnel manipulating the Sterne 34F_2_ are not greater than those posed by other category 2 organisms (Centers for Diseases Control and Prevention, Division of Foodborne, Bacterial and Mycotic Diseases, 2009); (iii) high-quality samples are not required, the anticomplementary reactions occurring rarely; (iv) the CFT is not animal species-specific: the same reagents are used to test sera from a variety of animal species and humans.

We did not test serum samples from anthrax-vaccinated people: however, since the procedure for detection of animal antibodies are identical to those used for human antibodies, we think that the Sterne-based CFT could be a useful research tool to evaluate the immunogenicity of anthrax vaccines in humans. This methodology, if officially accepted, could replace the lethal challenge with virulent anthrax strain, which is an expensive and increasingly unacceptable test.

The Sterne-based CFT could be particularly useful in veterinary medicine since serological tests are mostly involved in testing of herds or flocks of animals rather than single animals; the analysis of serological data should indicate whether the herd/flock is infected/vaccinated or not to obtain epidemiological information.

The CFT results indicated that the production of specific antibodies induced by vaccination with Sterne 34F_2_ did not persist over a long period of time in cattle. In many studies, conducted in cattle and goats, the antibody response following vaccination against anthrax was monitored using a PA-based ELISA, currently accepted as the best serological procedure, and different antibody kinetics were observed (Dipti et al., 2013; Roy et al., 2013; Hassan et al., 2015). However, the comparison of serological data is very difficult since many factors affect the immune response of animals, such as the vaccine type, the age of vaccination and, mostly, the health status of animals (IOWA Beef Center website, 2015).

The importance of antibody kinetics in relation to the vaccine efficacy has been well demonstrated; it is a crucial information that should be taken in consideration for the implementation of effective prevention and control measures to protect population, also considering that *B. anthracis* is one of the preferred pathogenic agents for use as bacteriological weapon in bioterrorism attacks (Inglesby et al., 1999).

## Conflict of Interest Statement

The authors declare that the research was conducted in the absence of any commercial or financial relationships that could be construed as a potential conflict of interest.
